# Local Targeted Therapy of Liver Metastasis from Colon Cancer by Galactosylated Liposome Encapsulated with Doxorubicin

**DOI:** 10.1371/journal.pone.0073860

**Published:** 2013-09-11

**Authors:** Chen Zhao, Qiang Feng, Zengpei Dou, Wei Yuan, Chenguang Sui, Xinghua Zhang, Guimin Xia, Hongfang Sun, Jie Ma

**Affiliations:** 1 State Key Laboratory of Molecular Oncology, Cancer Institute and Hospital, Chinese Academy of Medical Sciences, Peking Union Medical College, Beijing, P.R. China; 2 Abdominal Surgical Department, Cancer Institute and Hospital, Chinese Academy of Medical Sciences, Peking Union Medical College, Beijing, P.R. China; 3 Beijing National Laboratory for Molecular Science, College of Chemistry and Molecular Engineering, Peking University, Beijing, P.R. China; 4 Institute of Medicinal Biotechnology, Chinese Academy of Medical Sciences, Peking Union Medical College, Beijing, P.R. China; Complutense University, Spain

## Abstract

Since regional drug administration enables to maintain a high drug concentration within tumors, we compared the plasma concentration and biodistribution of doxorubicin (Dox) from drug-loaded conventional liposomes by local or systemic administration. The results demonstrated that drug concentration was substantially improved in liver as well as a decrease in blood and other organs by spleen injection mimicking portal vein perfusion (regional administration). To further investigate the targeted therapeutic effect of galactosylated liposome encapsulated doxorubicin (Dox) by regional administration, liver targeting liposomes were prepared by incorporating galactosylated-DPPE to conventional liposomes. Liposome uptake and targeting were verified *in vitro* and *in vivo* by fluorescence microscopy and xenogen IVIS imaging system, respectively. The results showed that galactose targeted liposomes presented a stronger specific cell uptake by human hepatocellular carcinoma HepG2 cells compared to the non-targeted liposomes. *In vivo* fluorescence imaging showed that the intra-hepatic deposition of conventional and galactosylated liposomes via spleen injection was more than that via tail vein administration, and galactosylated liposomes had higher fluorescent intensity over conventional liposomes in the liver post spleen administration. The anti-tumor effect of various drug administration routes for both liposomal formulations was evaluated using a murine liver metastasis model of colon cancer. The results indicated that tumor progression in the liver and mesenteric lymph nodes was significantly suppressed by Dox-loaded galactosylated liposomes via spleen injection, while no significance was observed in non-targeted formulations. Our data indicated that local perfusion of galactosylated liposomal doxorubicin had a great promise for the treatment of liver metastasis from colon cancer.

## Introduction

Following lymph nodes, the liver ranks the second organ with high incidence of metastasis. According to statistics, there are more than 70% of patients presenting liver metastases with their primary cancers located at the colon, lung, bone or brain [1], [2]. Metastasis is regarded as one of the most important prognostic factors for cancer patients. For example, the 5-year survival rate of gastric cancer with liver metastasis is less than 10% [3], [4], [5], [6].

Despite recent improvements in first-line chemotherapeutic strategies for the treatment of patients with liver metastasis from primary cancer, liver resection offers the only cure opportunity [7]. However, approximately only 15% of patients with liver metastasis are resectable [8], the additional 30% need chemotherapy before resection [9], [10], [11], [12]. It is, therefore, necessary to develop more effective regimens to prolong the survival of patients with liver metastases from original cancers.

Composed of transcatheter hepatic arterial chemoembolization (TAE) and portal vein chemotherapy, interventional chemotherapy has been considered a promising therapeutic treatment for hepatic metastatic carcinoma [13], [14]. Compared with systemic chemotherapy, regional drug administration via either arterial infusion or portal vein enables to maintain a high drug concentration and provide high levels of cytostatic activities within the tumor [15]. It is reported that regional intra-arterial chemotherapy has improved the response rate and life quality of patients with liver metastases from colorectal cancers [16]. This demonstrated that the interventional treatment is an effective way to stable the progression of liver metastases.

As one of commonly used agents for interventional therapy, doxorubicin has severe systemic side effects such as cardiotoxicity and bone marrow suppression, which limit its clinical use. This deficiency may be overcome by methods increasing drug accumulation within the tumor or using effective drug carrier that can deliver and release its cargo at the target site. Liposomes, the most widely investigated drug delivery system, have high accumulation in the liver while low immunogenicity, biocompatibility and drug protection [17]. Furthermore, liposomes conjugated with specific ligands could modify the pharmacokinetic characteristics and tissue distribution profile of drugs. This may lead to enhance efficacy as well as reduced toxic side-effects of antitumor drugs. Located in the mammal hepatocytic membranes, asialoglycoprotein receptor (ASGPR) may mediate the specific binding of galactoside-containing liposomes to hepatocytes [18], [19]. Such a liposome modified with *β*-D-galactose may provide significant therapeutic benefits to tumors in liver due to active targeting. In addition, liposomal carriers can exhibit a higher specificity towards malignant tumor tissues because they can theoretically leak out of the blood vessels in the tumor surrounding. This phenomenon has been known as the Enhanced Permability Retention (EPR) effect, a.k.a. passive targeting [20]. Therefore, it is reasonable to assume that the employment of Dox-loaded galactosylated liposomes (both passive and active targeting to intrahepatic tumors) in interventional chemotherapy (local administration to enhance the drug concentration) may improve therapeutic outcome for patients with liver metastasis and, at the same time, decrease systemic side effects of the drug.

In this study, we proposed a new therapeutic strategy that combined organ targeting drugs (galactosylated liposomal Dox) with regional administration. The purpose was not to target Hepatocellular carcinoma (HCC) but the liver, which might lead to a broad range of treatment for metastasis of different tissue origin in liver. Since there are abundant asialoglycoprotein receptor expressed on hepatocyte, galactosylated liposome was selected as the mediator to target the liver. Although the anti-tumor effect of drug-loaded galactosylated liposomes on liver cancer has been demonstrated in adequate preclinical models, no studies of its application in metastatic liver tumors have been reported. As blood from both intestines and spleen is normally carried to the liver through the portal vein, the spleen injection was selected to mimic portal vein perfusion to evaluate the advantages of galactosylated liposomal Dox in the treatment of hepatic metastatic carcinoma. Before the application of targeted liposomes in regional administration, the plasma concentration and biodistribution of doxorubicin from drug-loaded conventional liposmes were determined to evaluate the superiority of local over systemic administration for the increase of the drug concentration in target organ. Subsequently, the anti-tumor effect of the novel therapeutic strategy for colon cancer with liver metastasis was investigated using an animal model of hepatic metastasis from colon cancer. To our knowledge, this would be, to date, the first study on such therapeutic strategy. Our results may provide an alternative for patients with unresectable liver metastasis from primary tumors.

## Results

### The Pharmacokinetics Studies for Dox-loaded Conventional Liposomes

**Figure 1:**
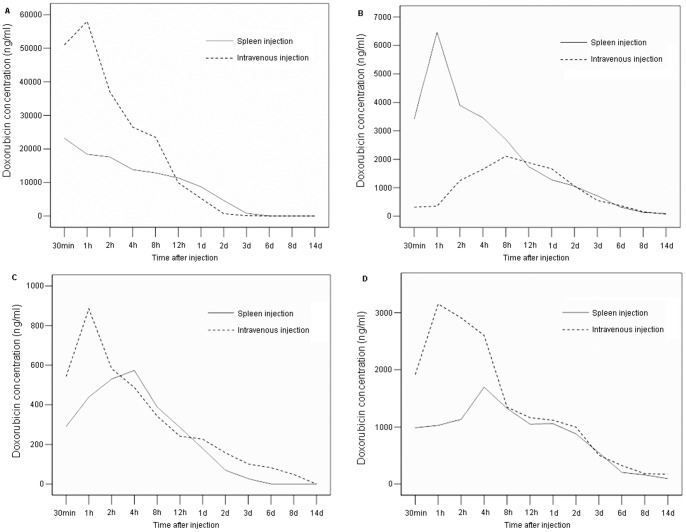
Doxorubicin concentration in plasma, liver, heart and kidney. Liposomal doxorubicin at a loading dose of 6 mg/kg was introduced into Balb/c-nu mice via either spleen injection or i.v. injection. After drug administration, the blood, liver, heart and kidney were collected from treated mice. Doxorubicin concentrations versus time in plasma (A), liver (B), heart (C) and Kidney (D) were determined by HPLC.

To evaluate the effect of various administration routes on the concentration and biodistribution of drugs from liposomal carriers, the pharmacokinetics studies for Dox-loaded conventional were performed. Liposomal doxorubicin at a loading dose of 6 mg/kg was introduced into Balb/c-nu mice via either spleen injection or i.v. injection. As shown in figure 1, the peak plasma concentration of Dox from spleen injection group was significantly lower than that from i.v. injection group (p<0.01). Twelve hours post injection, the plasma concentration of drug from both groups became similar. Furthermore, the drug concentration was substantially enhanced in liver within 10 hrs post local administration compared to i.v. injection (p<0.01), while it was lower in heart and kidney. This unique pharmacolinetics profile may lead to extended clinical benefits resulting in an improved therapeutic effect to intrahepatic tumors and a reduced side-effect to other organs. These results indicated that the tentative idea of liposomal carriers combined with local administration for the therapy of liver metastatic tumors was feasible. Based on this result, we further designed the new therapy regimen in this study that combined targeted liposomes with spleen injection to investigate if it could arise better anti-tumor effect. To achieve this, galactosylated liposomes were prepared by incorporating galactosylated DPPE to conventional liposomes.

### The Characterization of Galactosylated DPPE (Gal-DPPE)

To prepare galactosylated liposomes, one of the excipients, galactosylated DPPE, was first produced. The scheme for synthesis of Gal-DPPE was shown in supporting information (Figure S1). The intermediate product 5 (indicated in Figure S1) has been identified by the ^1^H NMR (400 MHz, CDCl_3_): δ 4.97 (br s, 1H, H-1), 4.07–4.03 (m, 2H), 3.95–3.93 (m, 1H), 3.81 (m, 1H), 3.72–3.64 (m, 3H), 3.57 (m, 1H), these 8Hs included the -CH- and -CH_2_- in galactose (except H-1) and -OCH_2_-, 2.79 (s, 4H, -COCH_2_CH_2_CO-), 2.39–2.38 (t, 2H, -CH_2_COO-), 1.61–1.58 (m, 4H, -OCH_2_C*H*_2_- and -C*H*_2_CH_2_COO-), 1.32 (m, 8H, 4×-CH_2_-) ppm.

The final product Gal-DPPE has been well characterized as follows. ^1^H NMR (400 MHz, CD_3_OD): δ 5.36–5.32 (m, 1 H,-CHOCOR), 5.30–5.18 (br s, 1 H, H-1), 4.04–3.34 (m, 16 H, -CH_2_OCOR, 2×−CH_2_OP-, -OCH_2_-, -CH_2_N- and the -CH- and -CH_2_- in galactose except H-1), 2.35–2.11 (m, 6 H, 2×-CH_2_COO-, -CH_2_CON-), 1.74–1.50 (m, 8 H,-C*H*_2_CH_2_CON-, 2×−C*H*_2_CH_2_COO-, -C*H*_2_CH_2_O-), 1.44–1.24 (m, 56 H, -CH_2_-), 0.93–0.86 (t, 6 H, 2×−CH_3_) ppm. This result was basically consistent with that in a previous report [21]. ESI-MS: m/z calculated for C_52_H_100_NNaO_15_P (M+H)^+^1032.7, found 1032.0.

### Liposome Characterization

Typically, all extruded liposome suspensions exhibited the particle size ranging from 100 to 140 nm, and the PDI of liposomes fell within the range of 0.1 to 0.2 (Table 1). The size distribution of each liposome was shown in supporting information (Figure S2). The diameter of galactosylated liposomes (Gal-lipo, 130.5 nm) was slightly larger than that of conventional liposomes (CL, 106.2 nm) due to the incorporation of Gal-DPPE. Both Dox-loaded CL (114.9 nm) and Gal-lipo (134.8 nm) presented a slightly higher z-average diameter values than those of empty liposomes resulting from the encapsulated doxorubicin molecules. Nevertheless, the results indicated that liposomes prepared in this study could deliver the drug to liver since the size of endothelial fenestrae in mouse liver sinusoids is over 140 nm [22].

**Table 1:** The Z-Average diameter of liposomes before/after drug loading.

	Z-Average (d. nm)	Polydispersity index (PDI)
Conventional liposome (CL)	106.2	0.11
Galactosylated liposome (Gal-lipo)	130.5	0.146
Dox-loaded CL	114.9	0.136
Dox-loaded Gal-lipo	134.8	0.185

### Liposome Uptake Analysis

**Figure 2:**
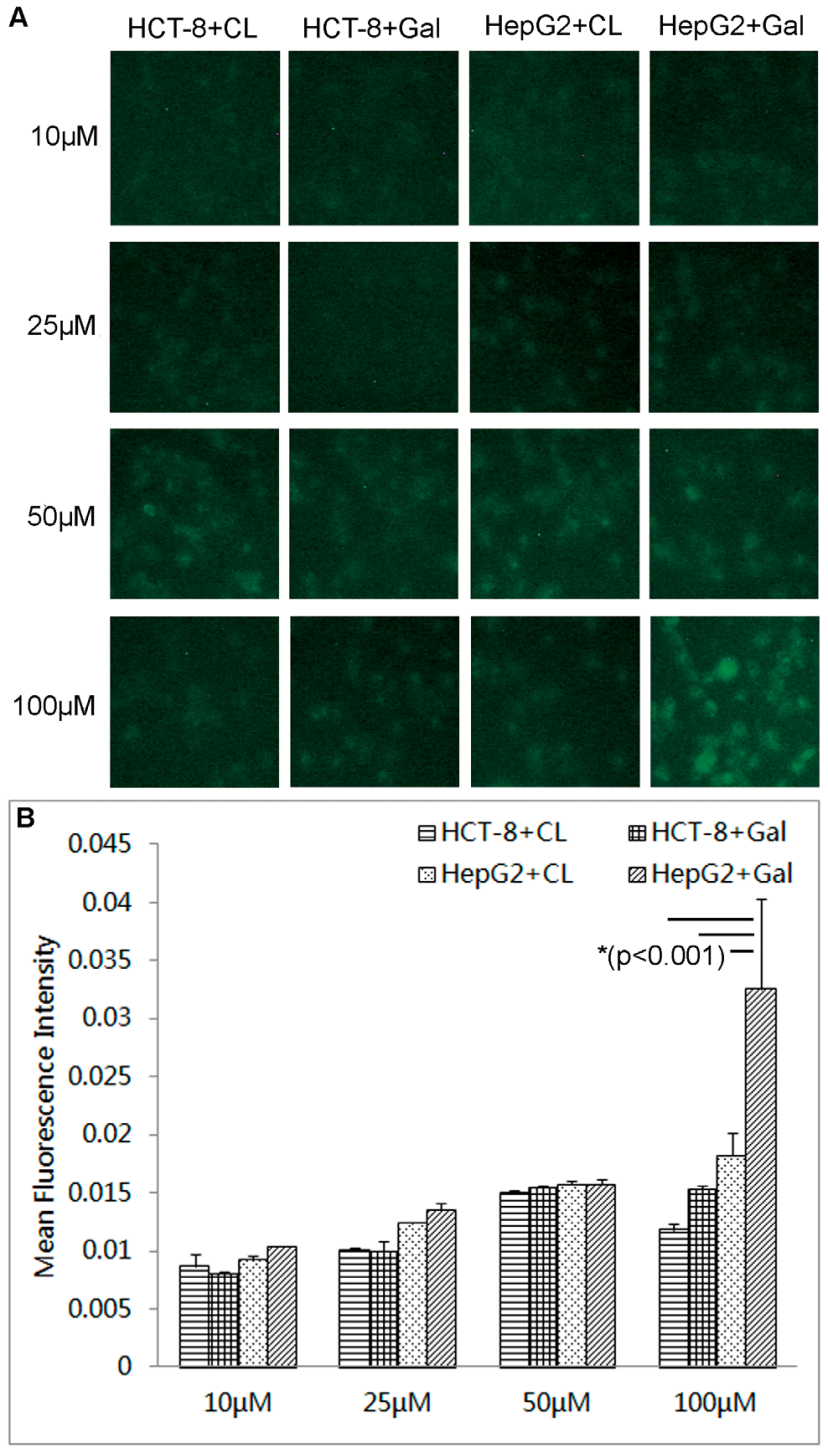
The effect of liposome concentration on cell uptake by ASGPR+/− cells. The HCT-8 cells (ASGPR−) and HepG2 cells (ASGPR+) were incubated with either conventional liposomes (CL) or galactosylated liposomes (Gal-lipo) for 2 hrs at different lipid concentration ranging from 10 µM to 100 µM. Cellular uptake of liposomes was visualized under fluorescence microscopy due to the incorporation of 25-NBD-cholesterol into liposomes. The mean fluorescence intensity presented by cells was determined using Image-Pro Plus Imaging software (Media Cybernetics). A) Fluorescence imaging of cellular uptake of liposomes. B) Quantitative analysis of the mean fluorescence intensity. “*” indicates significant difference (p<0.001). CL: conventional liposomes; Gal: galactosylated liposomes.

The effect of liposome concentration on uptake by ASGPR^+^ HepG2 cells and ASGPR^−^ HCT-8 cells are shown in Fig. 2. The mean fluorescence intensity of cells after 2 hrs incubation with liposomes was determined using Image-Pro Plus Imaging software (Media Cybernetics) for quantitative evaluation. The differences of fluorescence between the two cell lines incubated with respective liposomes could not be distinguished when the lipid concentration was below 100 µM (seen Figure 2B), although HepG2 cells treated with Gal-lipo presented slightly higher fluorescence intensity than the others. This might be ascribed to high level of nonspecific cellular uptake when lipid concentration was low. The specific binding was thus not enough to display the significant differences between groups. With the increased concentration of Gal-lipo (100 µM), the HepG2 cells could be clearly stained under the fluorescence microcopy at Ex/Em of 488 nm/505 nm, showing significantly higher cell uptake compared with the fluorescence intensity presented by HCT-8 cells treated with either Gal-lipo or CL or HepG2 cells treated with CL at the same lipid concentration (p<0.001). This indicated that specific uptake of Gal-lipo by ASGPR^+^ HepG2 cells was elevated with the increasing lipid concentration to a level of significant difference. The cellular uptake of HepG2 and HCT-8 cells incubated with 100 µM of various liposomes for 4 hrs was also observed, and no differences were found between groups (data not shown). This suggested that nonspecific uptake would be greatly improved when incubation time was long enough, resulting in the reduced difference of fluorescence produced by specific uptake. The concentration of 100 µM of lipid could ensure sufficient fluorescence signals to reflect the effect of receptor-mediated liposome uptake during 2 hours. Thus, 100 µM of lipid concentration was selected for the following experiments.

**Figure 3:**
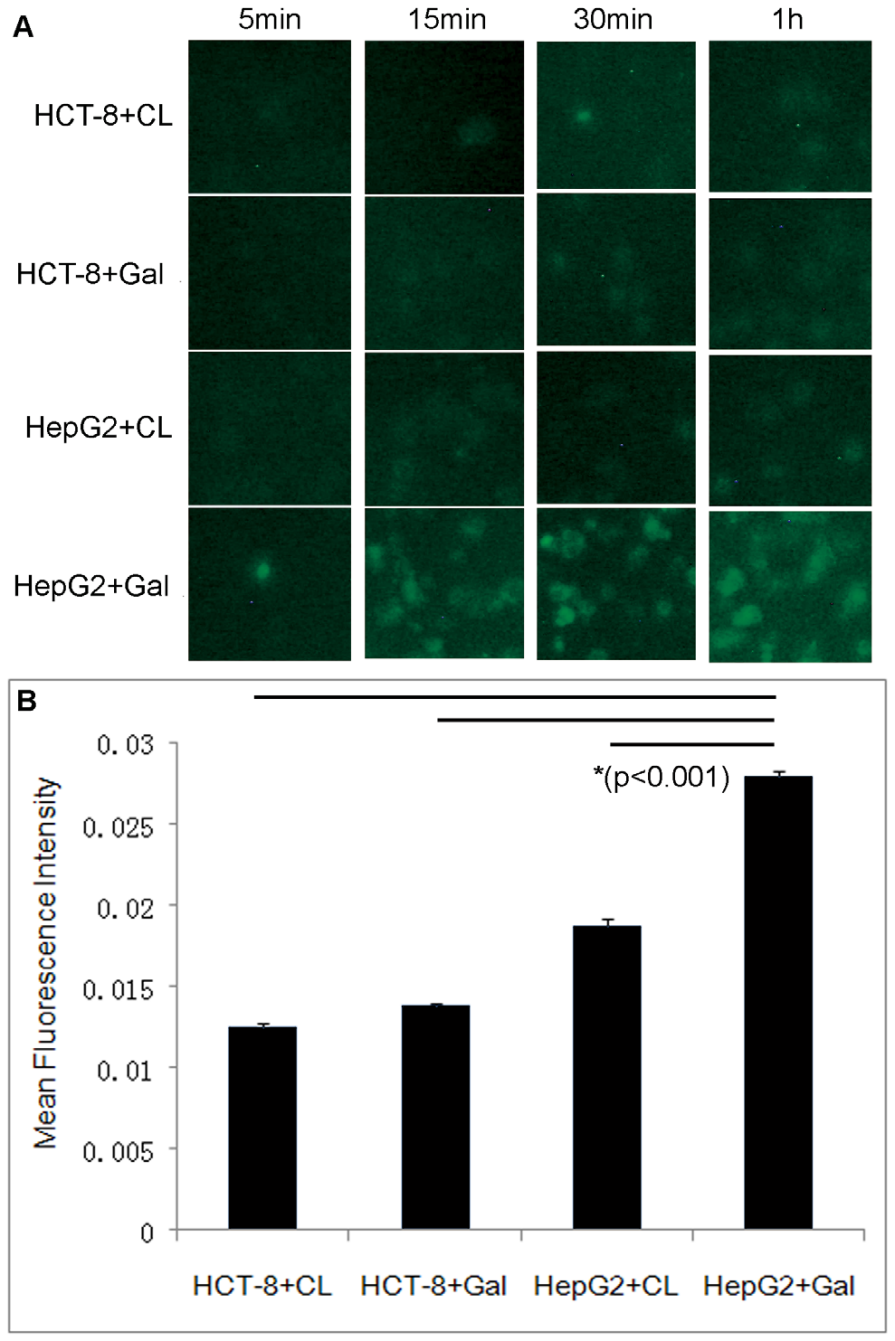
The effect of incubation time on cell uptake by ASGPR+/− cells. The HCT-8 cells (ASGPR−) and HepG2 cells (ASGPR+) were incubated with either conventional liposomes (CL) or galactosylated liposomes (Gal-lipo) at 37°C for different time duration. Cellular uptake of liposomes labeled by 25-NBD-cholesterol was visualized under fluorescence microscopy. The mean fluorescence intensity representing durg uptake was determined using Image-Pro Plus Imaging software. A) Fluorescence imaging of cellular uptake of liposomes. B) Quantitative analysis of the mean fluorescence intensity for the treatment of 1 hr incubation. “*” indicates significant difference (p<0.001). CL: conventional liposomes; Gal: galactosylated liposomes.

For the assessment of specific cell uptake, HCT-8 and HepG2 cells were incubated with CL/Gal-lipo for different time duration, respectively. Basically, incubation of NBD-cholesterol incorporated liposomes with cells at 37°C resulted in a time-dependent uptake (Fig. 3). HepG2 cells treated with Gal-lipo presented a minor uptake within 15 min of incubation, while no green fluorescence was observed when HepG2 cells incubated with CL. Such fluorescence became mounting at 30 min and 1 hr incubation. The mean fluorescence intensity of HepG2 cells incubated with Gal-lipo for 1 hr was markedly stronger than those of other treated groups (p<0.001), indicating higher cell uptake. On the contrary, the HCT-8 cells incubated with either CL or Gal-lipo hardly induce any cell uptake at all incubation time period. At 2 hrs incubation point, no significantly enhanced intensity of fluorescence was observed in Gal-lipo treated cells comparing with that of 1 hr incubation (Data not shown). Therefore, 1 hr of incubation was considered to be sufficient to reflect specific cell uptake. This result well demonstrated that galactosylated liposomes could rapidly bind to ASGPR^+^ cells via the receptor but not the ASGPR^−^ cells, which is a more effective way for the uptake of galactose-targeted liposome than pinocytosis.

**Figure 4:**
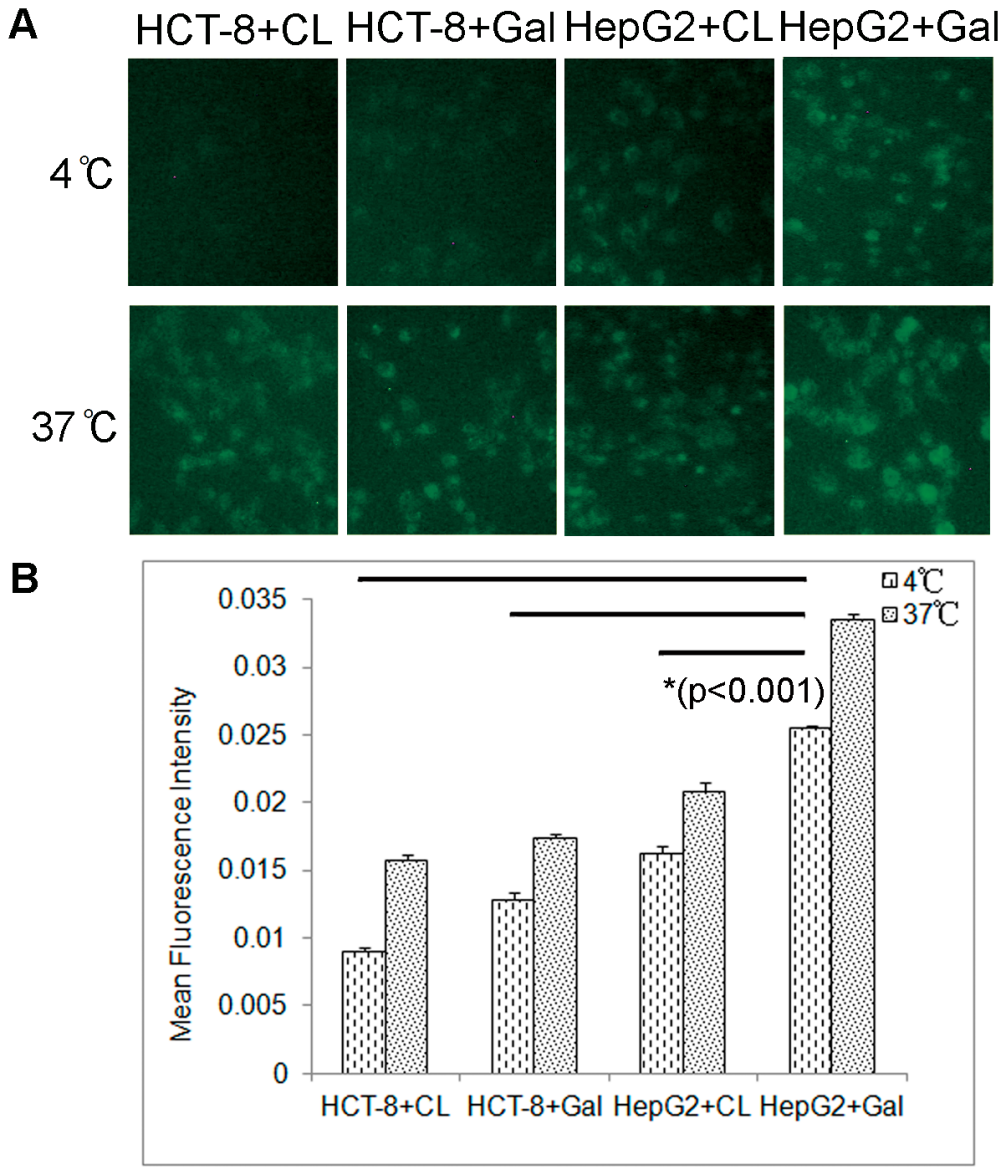
The effect of temperature on cell uptake by ASGPR+/− cells. The HCT-8 cells and HepG2 cells were incubated with either conventional liposomes (CL) or galactosylated liposomes (Gal-lipo) at 4°C for 1 hr, and then warmed to 37°C with continued incubation for an additional 1 hr. Cellular uptake of liposomes labeled by 25-NBD-cholesterol was visualized under fluorescence microscopy. The mean fluorescence intensity was quantitatively determined using Image-Pro Plus Imaging software. A) Fluorescence imaging of cellular uptake of liposomes. B) Quantitative analysis of the mean fluorescence intensity. “*” indicates significant difference (p<0.001). CL: conventional liposomes; Gal: galactosylated liposomes.

The nonspecific internalization of liposomes by cells via endocytosis may be reduced due to the weak fluidity of membrane, which could be caused by the changes of the rigidity of the lipid bilayer, its functionality and/or the level of cellular energy under low temperature [23]. However, receptor mediated binding between target cells and ligand-conjugated liposomes can occur at both 4°C and 37°C [24]. This suggested that receptor-mediated endocytosis would be the primary pathway for cellular uptake of liposomes under a low temperature condition. Therefore, the targeting of galactosylated liposomes was further investigated on ASGPR^+/−^ cells at different incubation temperature. As illustrated in Fig. 4, both CL and Gal-lipo failed to transport fluorescence to HCT-8 cells after 1 hr incubation at 4°C under fluorescence microscopy. The HepG2 cells, however, showed relatively weak but visible green fluorescence after the incubation with Gal-lipo for 1 hr at 4°C. Furthermore the mean fluorescence intensity from Gal-lipo treated HepG2 cells was significantly stronger than that from CL treated cells or Gal-lipo treated HepG2 cells (p<0.001). This indicated that Gal-lipo could target HepG2 cells and receptor-mediated endocytosis contributed to the obviously increased cellular uptake of targeted liposomes. Upon raising the temperature to 37°C, weak fluorencence of all the control group cells could be observed. Furthermore, HepG2 cells incubated with Gal-lipo represent stronger fluorescence intensity than that at 4°C. These data suggested the nonspecific cellular uptake of liposomes would occur at body temperature.

### Distribution Profile of CL and Gal-lipo

**Figure 5:**
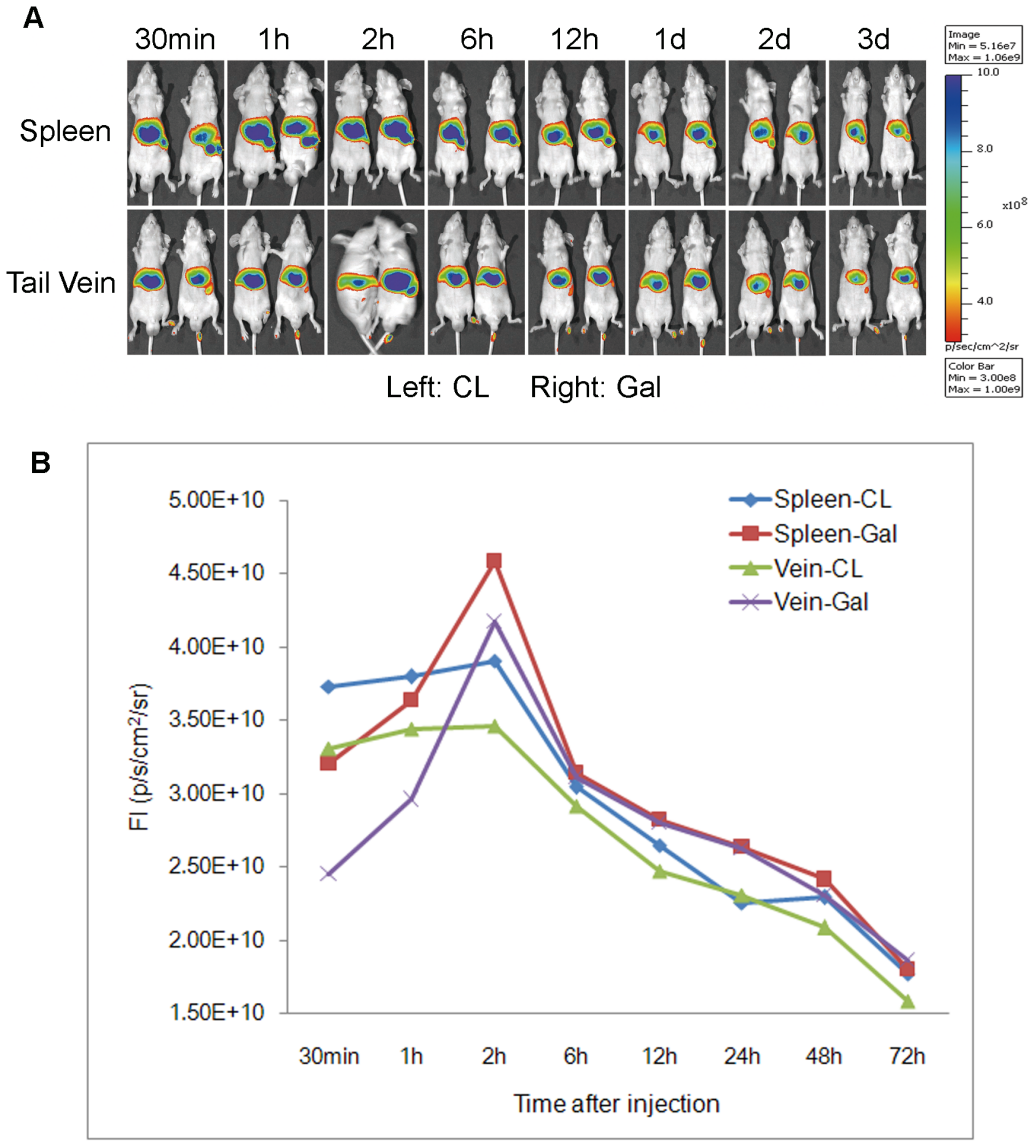
Distribution profiles of CL and Gal-lipo in the liver of mouse. The conventional liposomes (CL) and galactosylated liposomes (Gal-lipo) were labeled by DiR. Each liposome containing a total of 200 µg of lipid was ininjected into Balb/c-nu mice via either tail intravenous or spleen administration. The *in vivo* biodistribution was monitored by a live animal imaging system with Ex/Em of 745 nm/820 nm at various time point post injection. The photon radiance on the surface of the liver of an animal was expressed as photons per second per square centimeter per steradian (p/sec/cm^2^/sr). Images are compound pictures generated by Living Image software. A) In vivo longitudinal monitoring of both liposomes. B) The analysis of fluorescence imaging using Living Image software.

To investigate the targeting ability of Gal-lipo, both liposomes were labeled by a lipophilic fluorescent dye DiR and introduced into mice by i.v. and spleen injection, respectively. The *in vivo* biodistribution and real-time trafficking of CL and Gal-lipo were visualized using living fluorescence imaging technology. As it would cost several minutes to finish the spleen injection, only one mouse was included in each group. However, this experiment was performed for three times, and a representative result was shown in Fig. 5. It was found that the liposomes were predominantly deposited in liver and spleen regardless of liposomal composition and administration route (Fig. 5A). The intra-hepatic fluorescent intensity was gradually increased in all treated mice and the peak was achieved at 2 hrs post liposomal administration followed by a decline in a time-dependent manner (Fig. 5B). The red fluorescence in liver from galactosylated liposomes did not present the dominance at the first observation time point (30 min post injection). Conversely, it was lower than that from conventional liposomes via both tail vein and spleen injections. However, the intra-hepatic fluorescent intensity of Gal-lipo was rapidly increased in 1 hr and exceeded that of CL for both administration routes, indicating that more galactosylated liposomes were accumulated in liver than CL. In addition, the fluorescent intensities from both liposomes via spleen injection were maintained at higher levels than those via tail vein injection, indicating that the intra-hepatic deposition of liposome could be improved by spleen injection method. Furthermore, the strongest fluorescence was observed on the mouse injected with Gal-lipo via spleen at 2 hrs post injection, suggesting that both organ targeting of the carrier and regional administration route could enhance the accumulation of drug delivery system (liposome) in liver, which could effectively improve the drug concentration in target organ.

### *In Vivo* Studies

The objective of this study is to develop a novel therapeutic regimen for the intervention treatment of metastatic liver cancer. The strategy is to target organ–the liver, but not cancer cells. Therefore, an artificial liver metastatic model of colon cancer was established using human colon cancer cell HCT-8. Since HCT-8 cells have high magnitude of malignancy, the inoculated tumor in the liver could further metastasize to mesenteric lymph node. The progression of tumor was evaluated by two points: 1) AS the tumor in liver presented irregular shape, it is difficult to measure tumor size accurately. The weight of tumor in liver was hereby used to evaluate the hepatic tumor progression; 2) the weight of carcinoma in mesenteric lymph node. The mesenteric lymph node metastasis could be found in 2 weeks post intra-hepatic cell inoculation. Then the metastasis spread rapidly within 3–4 days and lead to the death of animals if no treatment proceeded. Therefore, all mice in this experiment received treatment using drug-loaded liposomes/free Dox on day 8 post cell inoculation and sacrificed on day 10 after drug administrations. The drug dose of 6 mg/kg was selected for *in vivo* treatment.

Though the method for portal vein injection in mouse has been reported [25], it remains to be a complicated surgery and hard to be effectively performed in a large number of animals. The spleen is a primary lymphoid organ which is known to stream into the liver via the splenic and portal veins [26]. Therefore, spleen injection was selected to mimic portal vein perfusion. This method had been proven to be effective for delivering liposome into the liver in our previous studies [27].

**Figure 6:**
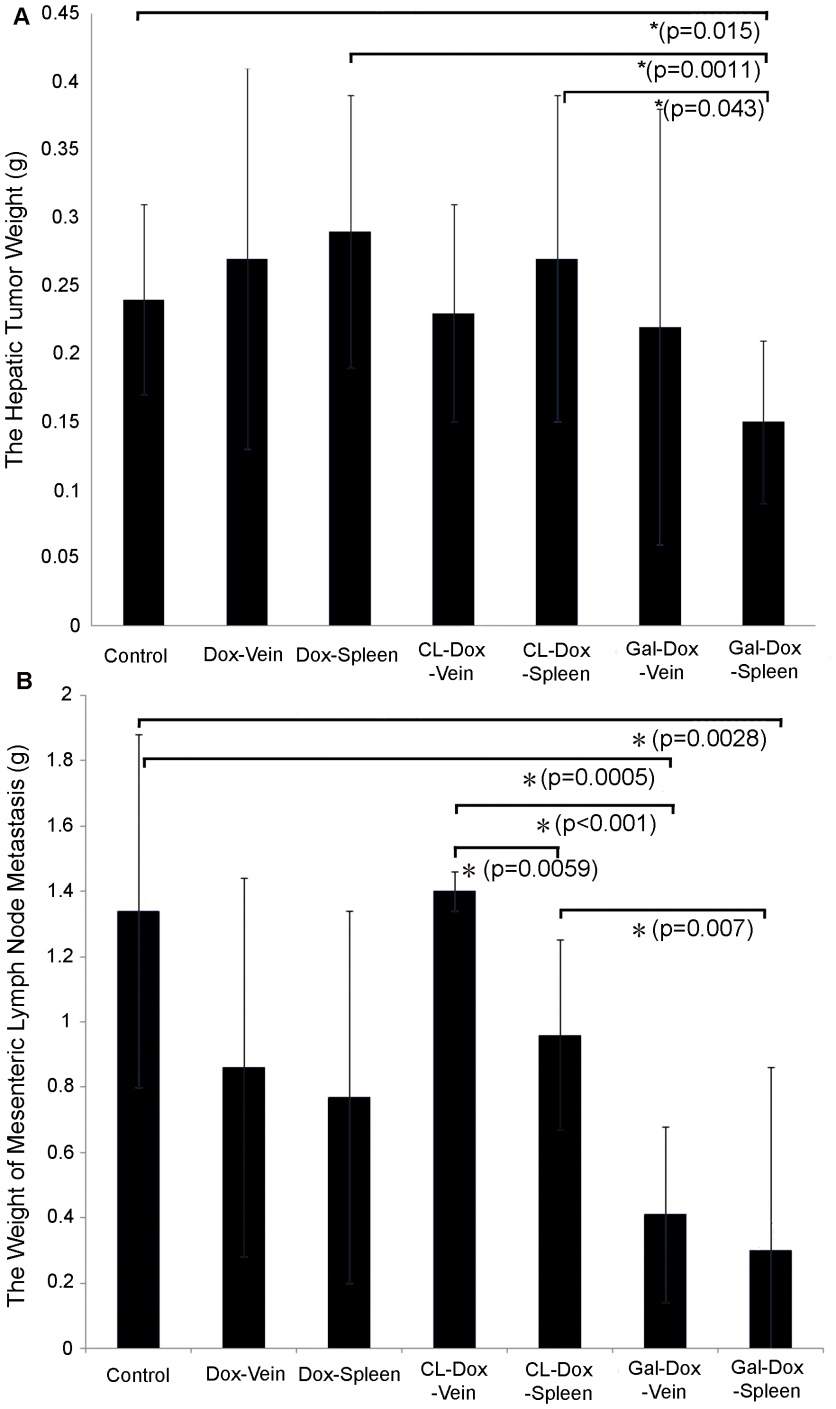
The anti-tumor effect of Dox-loaded galactosylated liposomes in animal model via spleen injection. Three formulations (Dox alone, CL-Dox and Gal-Dox) were introduced into tumor-bearing mice on day 7 post cell inoculation via two administration routes, tail vein injection and spleen injection. The drug dose administrated was 6 mg/kg. A) Tumor progression in liver was assessed by the mean value of hepatic tumor weight. B) Mesenteric lymph node metastasis was assessed by the mean weight of metastatic carcinoma from mesenteric lymph node. The results represent the mean ± SE. “*” indicate significant difference (p<0.05).

As revealed by the pharmacodynamics study (Fig. 6), treatment of free Dox had no therapeutic effects on hepatic tumor compared to the control of PBS, regardless of the route of drug administration. The mean hepatic tumor weight of free Dox treated groups via i.v. (0.27±0.14) and spleen injection (0.29±0.1) was comparable to that of PBS group (0.24±0.06). Similarly, CL-Dox resulted in marginal inhibition on hepatic tumor progression. The drug administration by both i.v. (0.23±0.08) and spleen injection (0.27±0.12) failed to effectively reduce the weight of tumor in liver compared to PBS group. However, drug-loaded Gal-lipo via spleen administration, as we expected, showed a significant effect in terms of the suppression of tumor progression in the liver (0.15±0.06, p_Gal/spleen vs PBS_ = 0.015), although such liposomal formulation via i.v. played little role in the inhibition of hepatic tumor progression (0.22±0.16). Furthermore, marked tumor inhibition could be observed in the group treated with drug-loaded Gal-lipo via spleen over that with free Dox (p_Gal vs Dox/spleen = _0.0011) or CL-Dox (p_Gal vs CL/spleen = _0.043). This suggested that anti-tumor response was enhanced by targeted liposomes and local administration.

One of clinical indications in this tumor model is the metastasis of mesenteric lymph node, and thus it is an important indicator to assess tumor progression. The mean weight of carcinoma metastasizing to mesenteric lymph node from groups treated by free Dox via i.v. (0.86±0.57 g) or spleen injection (0.77±0.56 g) showed no significant difference compared to PBS group (1.34±0.54 g). Encapsulation of Dox in CL showed an even more severe metastasis than those treated by Dox. This may be ascribed to retardation of drug release from liposomes resulting in reduced free drug concentration at target site. Nevertheless, the mean weight of metastatic carcinoma from animals injected with CL-Dox via spleen was significantly lower than that via i.v. (p_CL/spleen vs i.v._ = 0.0059). This indicated that the route of spleen injection could improve the anti-tumor effect. Encouragingly, drug-loaded Gal-lipo via both i.v. (0.41±0.27, p_Gal/i.v. vs PBS_ = 0.0005) and spleen administration (0.3±0.5, p_Gal/spleen vs PBS_ = 0.0028) showed a considerably stronger effect in terms of the suppression of tumor progression in mesenteric lymph node compared with the control group. In addition, significant differences were found between animals treated with Gal-Dox and CL-Dox via either spleen injection (p_Gal vs CL/spleen_<0.001) or i.v. (p_Gal vs CL/i.v._ = 0.007) in the inhibition of metastasis of mesentery lymph node. These results demonstrated that therapeutic effect on metastasis from the liver could be greatly improved by galactosylated liposome via spleen administration mimicking portal vein perfusion.

**Figure 7:**
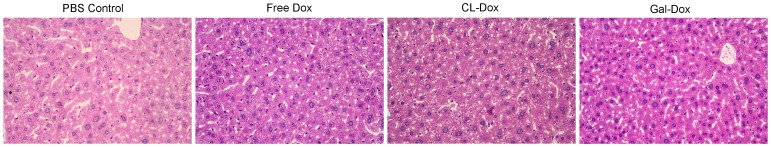
Microscopy images of H&E stained normal liver tissue slides. The normal liver tissues from groups treated with three drug formulations via spleen injection and PBS groups were collected and fixed using 10% formalin for 24 hrs at room, and then hematoxylin and eosin staining (H&E staining) was carried out. Magnification = 400×.

One of the most important reasons limiting the clinical application of Doxorubicin is its systemic side effects. Such systematic toxicity could be reduced by regional drug administration. Since the therapeutic strategy is to target the organ–liver, liver lesions potentially caused by the improved drug concentration resulting from local drug administration and targeted drug carrier (Gal-lipo) should be considered. Therefore, normal liver tissues of each tumor bearing mouse after the treatment with various drug formulations via spleen injection were collected and fixed using 10% formalin for 24 hrs, and H&E staining was performed. From the H&E staining results (Figure 7), no severe liver lesions could be observed among the samples from groups treated with free Dox, CL-Dox and Gal-Dox via spleen injection compared to PBS control group. Similar slight liver lesions were presented in these specimens, which could be ascribed to the inoculation of tumor cells in liver. In addition, no difference between CL-Dox and Gal-Dox group were observed. This result indicated that the regimen of targeting the organ would not cause the lesions of normal tissues but improve the anti-tumor effect although the drug concentration within liver was increased by organ targeted liposome and regional drug administration.

## Discussion

Cancer remains one of the most common leading causes of death. The development of effective therapeutic strategies is the highlight of biomedical research. As the malignancy of cancer is associated with their ability to form metastasis, the development of new methods to control the metastasis might be an alternative to improve the treatment outcome. To achieve this goal, we believe, two aspects should be considered. One is to increase effective concentration of anti-cancer drugs to destroy metastatic microenvironment of tumor and prolong the residence time of drugs. The other is to decrease side effects of drugs on normal tissues. Undoubtedly, liposomes have been proven to be an ideal drug carrier that has a strong impact on pharmacokinetics and tissue distribution of incorporated drugs resulting in enhanced efficacy as well as greatly reduced systematic toxicity of drugs. Moreover, regional chemotherapy such as portal vein perfusion generates a high drug concentration at the targeted site and simultaneously a low drug concentration in the systemic circulation and other tissues. To evaluate the potential application of liposomal formulations with local administration in the therapy of hepatic metastatic tumor, we first determined the plasma concentration and biodistribution of doxorubicin from drug-loaded conventional liposomes. The results showed that regional liposomal doxorubicin administration by spleen injection mimicking portal vein perfusion presented a significantly higher concentration in liver but lower in plasma, heart and kidney compared to systemic administration (i.v. injection). This indicated that local drug administration that we designed in this study could further improve the accumulation of drugs delivered by conventional liposomes in target organ–liver, and decrease possible toxicity to other organs simultaneously. Therefore, we believe, by the combination of liver-targeted liposomal formulations and regional drug administration, metastatic carcinoma from primary tumor should be suppressed more effectively. This study was designed to investigate the therapeutic effect of such a novel strategy using an animal model with implanted liver metastasis of colon cancer.

To improve the drug concentration in metastatic site–liver, galactosylated liposomes were prepared and used to deliver the anti-cancer drug. Since galactose residues can bind to the asialoglycoprotein receptor (ASGPR), an endocytotic cell surface receptor expressed by mammal hepatocytes, and trigger the import of molecules conjugated with galactose across the cellular plasma membrane [28], liposomes modified with galactosylate may target to hepatocytes via ASGPR-mediated way, thus provide significant therapeutic benefits to hepatic disease. The target ability of galactosylated liposomes was initially detected both *in vitro* and *in vivo*. The results of sufficient cell uptake by HepG2 cells demonstrated that the prepared Gal-lipos would predominantly bound to the asialoglycoprotein receptor (ASGPR) positive cells. In addition, our results showed that even if the cells were ASGPR negative, cell uptake for Gal-lipo was easier compare to that for conventional liposomes. This might be ascribed to the specific molecular configuration of Gal-DPPE, which facilitated nonspecific endocytosis. In the investigation of *in vivo* biodistribution, Gal-lipo via spleen administration exhibited the greatest accumulation in the liver at 2 hrs post injection, indicating its potential of delivering more drugs into the target site. Interestingly, the intrahepatic aggregation of Gal-lipo administered by either i.v. or spleen injection was initially lower than that of CL at 30 min post administration, and then enhanced rapidly to reach the peak at 2 hrs post injection, whereas CL increased steadily and started to decline at 2 hrs after administration. It has been proved that physicochemical properties, such as size, shape, and surface chemistry, can dramatically inﬂuence the behaviors of nanoparticles (NPs) in biological systems [29], [30], [31], [32]. For example, the targeting of liposomes and their anti-tumor effect after loading drugs might be altered due to the incorporation of large molecules such as the whole antibody [33]. The clearance time and subsequent biological organ deposition of NPs are dependent on the surface chemical modification (OH-, COOH- and PEG-) of particles [34]. In our study, Gal-lipo exhibited different characteristics than CL *in vitro* and *in vivo*. One possible contribution was the conformational differences between DPPE and Gal-DPPE conjugated with galactose by 8-(methoxycarbonyl)octanol linker.

Galactosylated liposomes encapsulating drugs have been documented in the treatment of liver cancer. Many investigators reported that liposomes modified with galactosylated lipid could specifically deliver their contents to liver, elevate drug concentration in liver and promote anti-tumor effect [35], [36], [37], [38]. However, very few studies, to our knowledge, about their application in liver metastasis from primary tumors have been reported. Many advanced out of liver cancers often develop hepatic metastasis, and most of them lack the expression of asialoglycoprotein receptors (ASGPR). Although galactosylated liposomes could not directly deliver the contents to tumor cells by receptor-mediated endocytosis, a large number of ASGPR on surounding hepatocytes (parenchymal cells) enable galactosylated liposomes to aggregate in the liver, resulting in significant organ targeting efficacy and particularly, enhancement of drug concentration around the metastasis. In our animal model, the liver metastatic tumor was established by the injection of HCT-8 colon carcinoma cells, which subsequently spread to mesenteric lymph node. The administration of Gal-Dox via spleen could effectively suppress both the hepatic tumor and the metastasis in the mesenteric lymph node compared with free Dox or CL-Dox. We speculated that this was resulted from the specific targeting of Gal-lipo leading to a relatively high drug concentration within the liver. *In vivo* biodistribution assay also confirmed that the aggregation of Gal-lipo occurred in the liver. These indicated Dox-loaded galactosylated liposomes could be an alternative method for the treatment of liver metastasis from other primary tumors.

Both animal and human studies have shown that liver tumors are primarily supplied by the hepatic artery [39], [40], while metastatic liver tumors by portal vein [41]. In this study, we created a new therapeutic strategy to apply drug-loaded galactosylated liposomes via spleen mimicking portal vein perfusion to improve the anti-tumor effect on metastatic liver tumors by increasing regional drug concentration and prolonging retention time. Approximately 75% of hepatic blood flow is derived from the hepatic portal vein, which drains blood from the gastrointestinal tract and spleen to capillary beds in the liver [42]. Thus, spleen administration, in this study, is supposed to be an alternative way to deliver prepared formulations in a mouse model. The pharmacokinetic studies relative to regional hepatic perfusion suggested that the drug concentration at the target site can be greatly increased [43], [44]. Our results on the plasma concentration and biodistribution of doxorubicin from CL also demonstrated the higher drug accumulation in liver and lower concentration in plasma and other organs. Therefore, we proposed a hypothesis that the application of liver target galactosylated liposomes in interventional therapy could further enhance the drug concentration in the liver. This is because: 1) liposomes, as a widely used drug delivery system, can passively target tissues or organs that have a discontinuous endothelium, such as the liver, spleen, and bone marrow [45]; 2) galactosylated liposomes can actively target the liver by specific binding to the ASGPR on hepatocytes. Although no significant differences of tumor inhibition for either hepatic tumor or metastasis in the mesenteric lymph node were found between groups treated with Gal-Dox via spleen and i.v., the anti-tumor effect was obviously better in spleen injection group because the weight of tumor in liver and mesenteric lymph node was substantially lower compared to that in PBS control, free Dox and CL-Dox groups. As spleen is a main organ that can take up liposomes, this may cause the retention in spleen of Gal-lipo, resulting in the reduced therapeutic effect. However, our results also demonstrated that the aggregation of Gal-lipo in the liver via spleen injection, a way mimmicing portal vein perfusion, was stronger than that via i.v., indicating that, to some extent, the drug concentration could be improved by the local administration. Thus, we believe this new therapeutic regimen would be applied to the interventional therapy for metastatic liver cancers.

Our research suggested that the improvement of anti-tumor effect on metastatic carcinoma in the liver probably resulted from the enhancement of drug concentration by organ targeted liposome and local drug administration. One worrisome problem is that the toxicity to the normal tissue of the targeted organ. The H&E staining slides from groups treated with various drug formulations via spleen administration showed that little lesion in the liver could be observed. This indicated that the new strategy would be a relative safe treatment for metastatic liver cancers.

The combination of doxorubicin with platinum-based anti-cancer drugs is the first-line chemotherapy protocol employed in interventional therapy for liver metastasis from colon cancer. Therefore, Dox was selected in our study. However, HCT-8 cells, which were used to establish animal tumor model, exhibited weak sensitivity to Dox treatment in our previous experiments (data not shown). A minimal dose of 30 µg/mL Dox was required to obtain over 70% of cell growth inhibition. Furthermore, this cell line presented severe malignancy as it developed both hepatic carcinoma and severe mesenteric lymph node metastasis with the inoculation of only 5×10^4^ cell in the liver. These might be the reason causing insignificant therapeutic effect by either free Dox or CL-Dox despite of spleen administration route. Moreover, direct cell killing by Dox-loaded CL may be delayed due to requirement of drug release from liposomes resulting in an insufficient naked drug concentration at the target site. On the contrary, both rapid accumulation of drugs on target site and steady and sustained elevation of drug concentration could be achieved by the targeting of galactosylated liposomes to the liver, and furthermore regional drug administration substantially increased the drug concentration up to a relatively high level. These greatly improved the inhibition of drug low-sensitive cancer cells.

In conclusion, galactosylated liposomes encapsulated with doxorubicin had stable physicochemical properties and carried a definite hepatic-targeting profile. A satisfactory efficacy has been achieved in the treatment of hepatic metastasis from colon tumors via portal vein chemotherapy. Such treatment regimens might become a promising strategy when dealing with patients with hepatic metastasis from other primary cancers.

## Materials and Methods

### Materials

Hydrogenated soybean phosphatidylcholine (HSPC), cholesterol (Chol), 25-[N-[(7-nitro-2-1,3-benzoxadiazol-4-yl)methyl]amino]-27-norcholesterol (25-NBD-cholesterol), 1,2-dipalmitoyl-*sn*-glycero-3-phosphoethanolamine (DPPE) were purchased from Avanti Polar Lipids Inc. (Alabaster, AL, USA). Doxorubicin hydrochloride was purchased from Shenzhen Main Luck Pharmaceuticals Inc (Shenzhen, China). Two cancerous cell lines, human colon cancer cell HCT-8 and human hepatocellular carcinoma cell line HepG2 were obtained from the American Type Culture Collection (ATCC, Rockville, MD, USA). The medium for cell culture Gibco® RPMI Media 1640 and the fluorescence dye 1,1′-dioctadecyl-3,3,3′,3′-tetramethyl indotricarbocyanine iodide (DiR) were purchased from Life Technologies Corp. (Carlsbad, CA, USA). 3-(4,5-dimethyl-2-thiazolyl)-2,5-diphenyl-2H tetrazolium bromide (MTT), dimethyl sulfoxide (DMSO) and other regular chemicals were purchased from Sigma-Aldrich Co. LLC. (St. Louis, MO, USA). All reagents, unless addressed, were of analytical grade and used as received.

### Synthesis of 8-[Carboxy-2-(1,2-dipalmitoyl-*sn*-glycero-3-phospho)ethanolamido] Octylgalactopyranoside (Gal-DPPE)

The scheme for the synthesis of Gal-DPPE was shown in Figure S1. All reagents were of analytical grade and dried before use. ^1^H NMR was recorded by a Bruker-400 MHz nuclear magnetic resonance spectroscopy (ARX400, Bruker Corporation, Switzerland). The molecular weight was measured by a Micromass ZAB-HS magnetic mass spectrometer (ZAB-HS, Micromass, Manchester, U.K.).

Gal-DPPE was synthesized following a previous report [21]. Briefly, 2,3,4,6-*Tetra*-*O*-benzoyl-galactopyranosyl trichloroacetimidate (indicated as product 1 in Figure S1) was synthesized by Becker [21]. The linker molecule, 8-(methoxycarbonyl)octanol (indicated as product 2 in Figure S1), was synthesized according to previous studies [46], [47]. Next, 8-(methoxycarbonyl)octylgalactopyranoside (indicated as product 3 in Figure S1) was synthesized based on the above two molecules with the aid of trifluoromethanesulfonic acid trimethylsilyl ester (Me_3_SiOTf) in CH_2_Cl_2_. Then the protected group was hydrolyzed by a two-step reaction (by the use of K_2_CO_3_/CH_3_OH and NaOH/H_2_O) to obtain 9-galactopyranosyloxy nonanoic acid (indicated as product 4 in Figure S1). Subsequently, *N*, *N*′-dicyclohexylcarbodiimide (DCC) and *N*-hydroxysuccinimide (NHS) were added to activate the carboxyl group and achieve *N*-(9-galactopyranosyloxy nonanoyloxy)succinimide (indicated as product 5 in Figure S1) as a white solid powder. This product was characterized by ^1^H NMR.

In the last, 25 mg product 5, 25 mg NaHCO_3_ and 20 mg 1,2-dipalmitoyl-*sn*-glycero-3-phosphoethanolamine (DPPE) were dissolved in 4 mL THF and 0.5 mL ddH_2_O. The mixture was stirred for 12 hrs at room temperature. After filtrated and evaporated under vacuum, the residue was freeze-dried, then purified on silica gel with CHCl_3_:CH_3_OH (4∶1) and on lipophilic Sephadex LH-20 with CHCl_3_:CH_3_OH (2∶1) to acquire the final product, 8-[Carboxy-2-(1, 2-dipalmitoyl-*sn*-glycero-3-phospho) ethanolamido] octylgalactopyranoside (Gal-DPPE), as a white solid powder (28 mg, 46%). The product was characterized by ^1^H NMR and ESI-MS.

### Preparation of Conventional and Galactosylated Liposomes

Two types of liposomes were prepared in this study, conventional liposome (CL) composed of HSPC, Chol and DPPE as well as galactosylated liposome (Gal-Lipo), comprised HSPC, Chol and Gal-DPPE. Both liposomes were prepared as previously described with minor modifications [48]. In brief, HSPC, cholesterol and DPPE/Gal-DPPE were dissolved in chloroform and added to a flask with a molar ratio of 2∶1: 0.1. Thereafter, the chloroform was removed under N_2_ and evaporated under vacuum for at least 1 hr to form a thin lipid film. A volume of 2 mL of ammonium sulphate (250 mM, pH 5.5) was added to the dried lipids. The lipid suspension was processed in a ultrasonic bath for 15 min at 65°C to form a milky solution of multilamellar vesicles, followed by extrusion at 65°C through a series of polycarbonate membranes with various pore diameters of 200 nm, 100 nm and 80 nm, respectively. The extrusion for each membrane was performed for 5 times. The particle size of the two liposomes was then measured using a Particle Analyzer, Zetasizer Nano ZS (Malvern Instruments Ltd, Worcestershire, U.K.).The phosphorus content for both liposomes was determined using the Bartlett phosphate assay [49]. Both types of liposome suspensions were stored in the fridge (0–4°C) and would be used within 1 week of the production.

### The Pharmacokinetic Studies for Dox-loaded Conventional Liposomes

Liposomal doxorubicin at a loading dose of 6 mg/kg was introduced into Balb/c-nu mice via either spleen injection or i.v. injection. For the splenic administration, liposomal suspension was directly injected into the parenchyma of the exposed spleen after the mouse was anesthesized by sodium pentobarbital. After the drug administration, the blood, heart, liver and kidney were collected from sacrificed mice at different time point. The plasma was separated by 5000 rpm centrifugation for 10 min. Tissue samples were processed using IKA T 10 basic (IKA-Werke GmbH & Co.KG - Staufen, Germany) after adding 1 ml of distilled water, and the supernatant were obtained by the centrifugation at 3000 rpm for 10 min. All specimens were stored at −20°C until use. Doxorubicin concentrations in plasma and other organs were determined by High-performance liquid chromatography (HPLC) using a chromatographic column Symmetry C18 (5 µm, 4.6×250 mm) and a waters 474 scanning fluorescence detector (excitation 487 nm, emission 593 nm), and a 30 µg/ml of doxorubicin solution was used as the internal standard. The mobile phase consisted of acetonitrile: 0.01 M NH_4_H_2_PO_4_ buffer, pH 3.0 (30∶70).

### Evaluation of Targeted Liposomes *in vivo* and *in vitro*

Two cancerous cell lines, human colon cancer cell HCT-8 and human hepatocellular carcinoma cell line HepG2, were used to assess the cell uptake of liposomes. The HepG2 cells contain asialoglycoprotein receptor (ASGPR) on their cell surface displaying a specific interaction with galactose. Such cells have been widely used to examine target specific drug delivery by galactosylated liposomes [50], [51], [52]. The HCT-8 cells, on the contrary, are ASGPR-negative, and thus believed to uptake liposomes by non-receptor mediated endocytosis. To observe cell uptake of both liposomes by ASGPR^+/−^ cells, 25-NBD-cholesterol was integrated into liposome bilayer at a molar ratio of 5%. The prepared liposomes could be visualized under fluorescence microscopy (Leica DMI 4000 B, Leica Microsystems Ltd. Beijing, China) with the excitation and emission wavelength of 488 nm and 505 nm, respectively, when internalized by cells. The mean fluorescence intensity presented by cells was determined using Image-Pro Plus Imaging software (Media Cybernetics) to quantitatively evaluate cell uptake.

The effect of lipid concentration on cell uptake was initially investigated to determine the optimal reaction condition for the observation of liposome uptake. Various concentrations of CL or Gal-lipo, ranging from 10 µM to 100 µM, diluted in Gibco® RPMI Media 1640, were incubated with HepG2 or HCT-8 cells seeded in a 96-well culture plate with the cell concentration of 1×10^4^/well at 37°C for 2 hrs. Subsequently, the cells were gently rinsed by phosphate-buffered saline (PBS) for 3 times to remove unbound liposomes and transferred to the fluorescence microscope for optical observation. Target-specific cell uptake was also examined by mixing two cells with two liposomes, respectively, and cultured at 37°C for different time duration to lessen the influence of non-receptor mediated endocytosis. To investigate the membrane fluidity and temperature effect on uptake, the cells were incubated at 4°C for 1 hr in the presence of fluorescence labeled liposomes (25-NBD) at 100 µM lipid concentration selected based on the result from the above mentioned assay and observed under the microscope. Then the temperature was elevated to 37°C with continued incubation for an additional 1 hr before microscope observation.

For assessment of the *in vivo* distribution of liposomes, a lipophilic tracer DiR, a long-chain dialkylcarbocyanine, was employed in this study. As the dye can uniformly label cells via lateral diffusion in the plasma membrane, it is, thus, reasonable to mark liposomes due to the similarity of their bilayer structure to that of the cell membrane. Fluorescence labeled liposomes were prepared by adding 10 µL of DiR to form a mixture containing 0.25 µM of dye. Each type of liposomes at a total lipid of 200 µg was introduced into Balb/c-nu mice via tail intravenous (i.v.) or spleen administration. The *in vivo* biodistribution was monitored by a live animal imaging system (IVIS 200; Xenogen, Hopkintown, MA) with Ex/Em of 745 nm/820 nm for DiR at certain time intervals. The photon radiance on the surface of an animal was expressed as photons per second per centimetre squared per steradian (p/sec/cm^2^/sr). Images are compound pictures generated by Living Image software (Caliper Life Sciences, USA).

### Establishment of Hepatic Metastatic Model

Animal studies were approved by Ethical Committee of Chinese Academy of Medical Sciences (CAMS) and Peking Union Medical College (PUMC) with approval ID SYXK2008-0025. All surgery was performed under sodium pentobarbital anesthesia and executed according to the legal requirements. All female Balb/c-nu mice, 6–8 weeks old with the weight of 18–20 g, were purchased from Institute of Laboratory Animal Sciences (ILAS) and housed in isolator cages. Human colon cancer cell line HCT-8 was used to establish the liver metastatic model. Briefly, the mouse abdomen was swabbed with 70% ethanol after anesthesized by pentobarbital sodium (50–60 mg/kg). The liver was exposured by cutting an abdominal incision near the xiphisternum. A total 5×10^4^ of HCT-8 cells in 1 µL PBS was injected into the liver of each mouse followed by surgical suture. Tumor growth was monitored by small animal ultrasound examination on days 7 and 17 post inoculation. The average sizes of tumor were approximately 2 mm on day 7 and 8 mm on day 17 in diameter, respectively.

### *In vivo* Treatment

The antitumor effect of both liposomal formulations (CL-Dox and Gal-Dox) via various routes of drug administration was evaluated on liver metastatic model derived from HCT-8. Seventy female Balb/c-nu mice were pre-inoculated with HCT-8 to establish the liver metastatic tumor. Three formulations (Dox alone, CL-Dox and Gal-Dox) were diluted in 5% glucose solution and then introduced into the mice with a drug dose of 6 mg/kg·mouse^−1^ via two administration routes, intravenous injection and spleen injection, on day 7 post cell inoculation. Therefore, there were 6 different treatment groups, named group A–F. Each group contained 10 tumor-bearing mice and the remaining 10 mice were received PBS injection only as control. All mice were sacrificed on day 10 post drug administration to assess the tumor progression. The hepatic and mesenteric lymph node tumor of each mouse were dissected carefully and weighed using an electronic balance measurement, respectively. The normal liver tissues from groups treated with three drug formulations via spleen injection and PBS groups were collected and fixed using 10% formalin for 24 hrs at room, and then hematoxylin and eosin staining (H&E staining) was carried out.

### Data Analysis

Analysis of data was performed with SPSS11.5 software (SPSS Science Products, USA). The data were expressed as mean±SEM. Error bars represent the standard deviation. The statistical assay was performed by Student’s *t*-test, and the differences were considered statistically significant only when p<0.05.

## Supporting Information

Figure S1 **Scheme for synthesis of Gal-DPPE.** The numbers in the figure represent various intermediate products.
(TIF)

Figure S2 **The size distribution of each liposome.** A) Conventional liposome (CL); B) Galactosylated liposome (Gal-lipo); C) Dox-loaded CL; D) Dox-loaded Gal-lipo.
(TIF)
