# Supplementary figures and images for: Local Targeted Therapy of Liver Metastasis from Colon Cancer by Galactosylated Liposome Encapsulated with Doxorubicin
Source: PLoS One. 2013 Sep 11;8(9):e73860. doi: 10.1371/journal.pone.0073860 (PMC3770687; doi:10.1371/journal.pone.0073860)

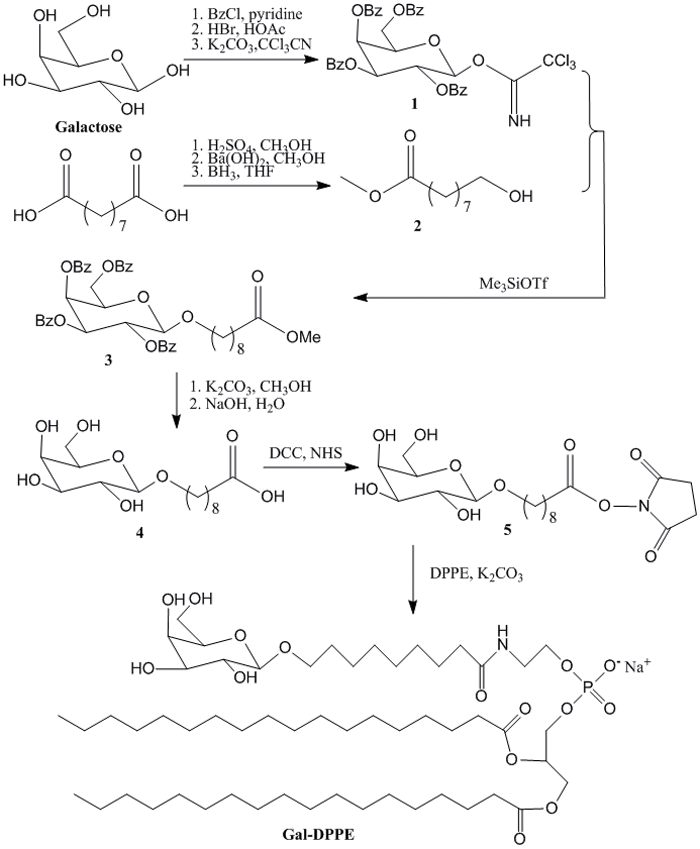

Supplement: Figure S1 — Scheme for synthesis of Gal-DPPE. The numbers in the figure represent various intermediate products. (TIF) [file pone.0073860.s001.tif]

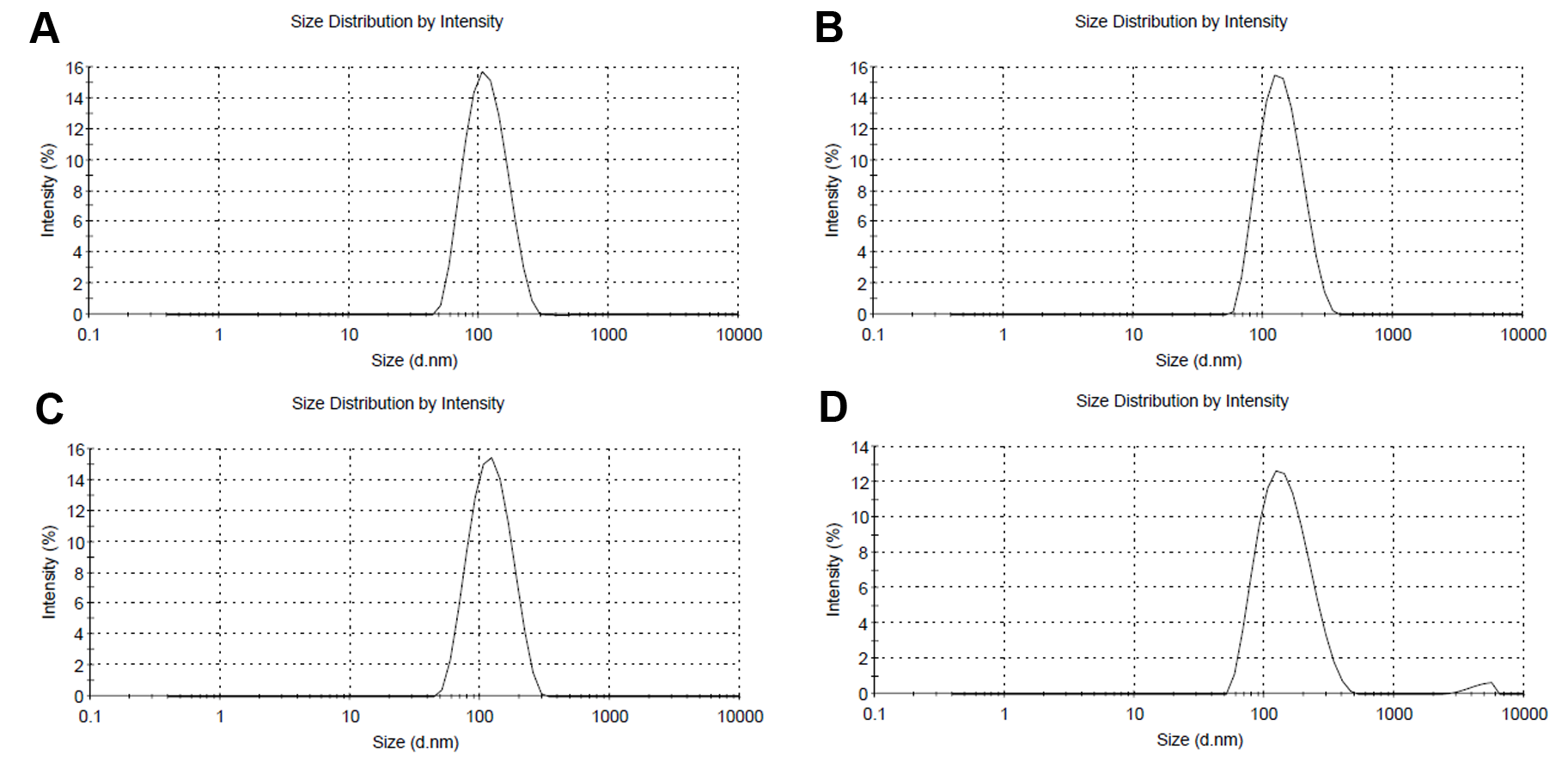

Supplement: Figure S2 — The size distribution of each liposome. A) Conventional liposome (CL); B) Galactosylated liposome (Gal-lipo); C) Dox-loaded CL; D) Dox-loaded Gal-lipo. (TIF) [file pone.0073860.s002.tif]
